# Protective effects of *Vitex agnus-castus *in ovariectomy mice following permanent middle cerebral artery occlusion

**DOI:** 10.22038/ijbms.2019.31692.7625

**Published:** 2019-09

**Authors:** Raheleh Alimohamadi, Iman Fatemi, Soudabeh Naderi, Elham Hakimizadeh, Mohammad-Reza Rahmani, Mohammad Allahtavakoli

**Affiliations:** 1 Student Research Committee, Rafsanjan University of Medical Sciences, Rafsanjan, Iran; 2 Research Center for Tropical and Infectious Diseases, Kerman University of Medical Sciences, Kerman, Iran; 3 Physiology-Pharmacology Research Center, Research Institute of Basic Medical Sciences, Rafsanjan University of Medical Sciences, Rafsanjan, Iran; 4 Department of Physiology and Pharmacology, School of Medicine, Rafsanjan University of Medical Sciences, Rafsanjan, Iran

**Keywords:** Anti-inflammatory, Mice, Ovariectomy, Stroke, Vitex agnus-castus

## Abstract

**Objective(s):**

Previous studies have indicated that phytoestrogens induce estrogenic as well as anti-inflammatory effects, and they are found in high abundance in the extracts of some herbs such as *Vitex Agnus Castus* (VAC). Therefore, we investigated the effect of VAC extract on ovariectomized mice after the induction of permanent middle cerebral artery occlusion (PMCAO) model.

**Materials and Methods:**

In this study, 50 mice ranging from 25 to 35 g were divided into five experimental groups as follows: Control, VAC, Estrogen, Tamoxifen, and Tamoxifen-VAC. Animals were ovariectomized, and after 30 days of treatment, they were given PMCAO induction. Behavioral assessment (adhesive removal and wire hanging tests) was evaluated 24 hr, 48 hr, and one week after induction of stroke. The infarct volume, as well as serum levels of matrix metalloproteinase-9 (MMP-9) and interleukin-10 (IL-10), were measured one week after stroke.

**Results:**

One week after stroke, in both VAC and estrogen groups, the infarct size reduced in comparison with the control group. Estrogen and VAC extract improved adhesive removal and wire hanging test, increased the level of IL-10, and decreased the level of MMP-9 compared with the control group. In addition, co-administration of tamoxifen and VCA extract had no significant effect on measured indices compared with control and tamoxifen groups.

**Conclusion:**

Based on our findings, VAC extract has neuroprotective properties and can reduce stroke injuries in PMCAO-induced ovariectomized mice via anti-inflammatory and estrogenic properties.

## Introduction

It has been demonstrated that administration of exogenous estrogen improves the outcomes in rodent stroke models (1). Estrogen replacement therapy in decreasing the cerebral ischemic damage and the expression of matrix metalloproteinase-9 (MMP-9) has been well established in animal models (2, 3). Also, previous studies have demonstrated that female hormones induce regulatory effects on the immunity system via elevating interleukin-10 (IL-10) levels (4, 5). On the other hand, increasing the level of IL-10 is related to the reduction of injury after middle cerebral artery (MCA) occlusion, which suggests the neuroprotective role of IL-10 in stroke (6). Despite its neuroprotection in human and rodent models of stroke, estrogen replacement therapy is accompanied by serious adverse effects such as breast and endometrial cancers as well as cardiovascular diseases (7). 

Similar to estrogen, phytoestrogens (exist in high abundance in some herbs) induce estrogenic and anti-inflammatory effects. The major sources of phytoestrogens are isoflavonoids such as isoflavones, coumestans, and lignans (8). These compounds show less estrogenic effect compared with estrogens and combine with estrogen receptors (ER) with different affinities (9). Many studies have provided evidence for the neuroprotective effect of phytoestrogens in numerous disorders, including stroke (10, 11). These phytoestrogens are thought to exert neuroprotection by either their agonistic effect on estrogen receptors (12) or induction of antioxidative, anti-inflammatory, and anti-apoptotic properties (13). Phytoestrogens inhibit the production of matrix metalloproteinase-9 (MMP-9) (14). On the other hand, some studies reported that phytoestrogens could dilate brain arteries and thereby improve cerebral blood flow (15, 16). This finding may suggest, at least in part, that neuroprotective action of phytoestrogen diets in animal models of ischemic stroke might be related to increased brain blood flow (11).

**Figure 1:**
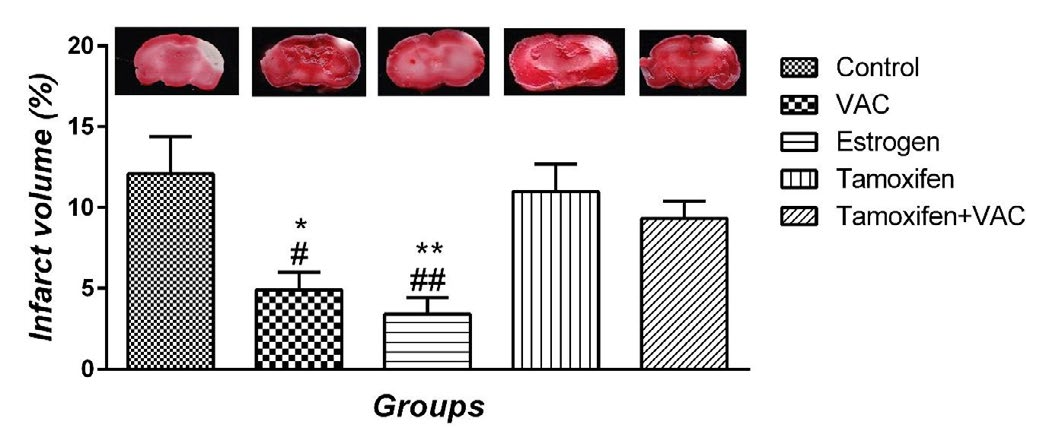
Assessing the effects of *Vitex agnus-castus* (VAC) extract, estrogen, tamoxifen, and the combination of tamoxifen plus VAC extract on infarct volume one week after stroke. Data are presented as means±SEM. *P<0.05 and **P<0.01 vs control group. #*P*<0.05 and ##*P*<0.01 vs tamoxifen group

**Figure 2:**
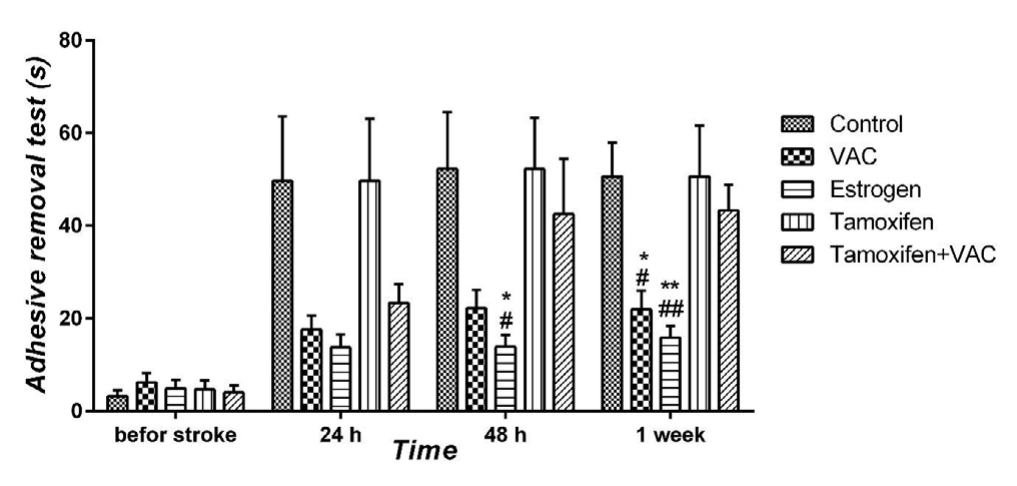
The effect of *Vitex agnus-castus* (VAC) extract, estrogen, tamoxifen and the combination of tamoxifen plus VAC extract on the sensorimotor function, which was measured by the adhesive removal test 24 hr, 48 hr, and one week after the stroke. Data are presented as means±SEM. *P<0.05 and **P<0.01 vs control group. #*P*<0.05 and ##*P*<0.01 vs tamoxifen group

**Figure 3:**
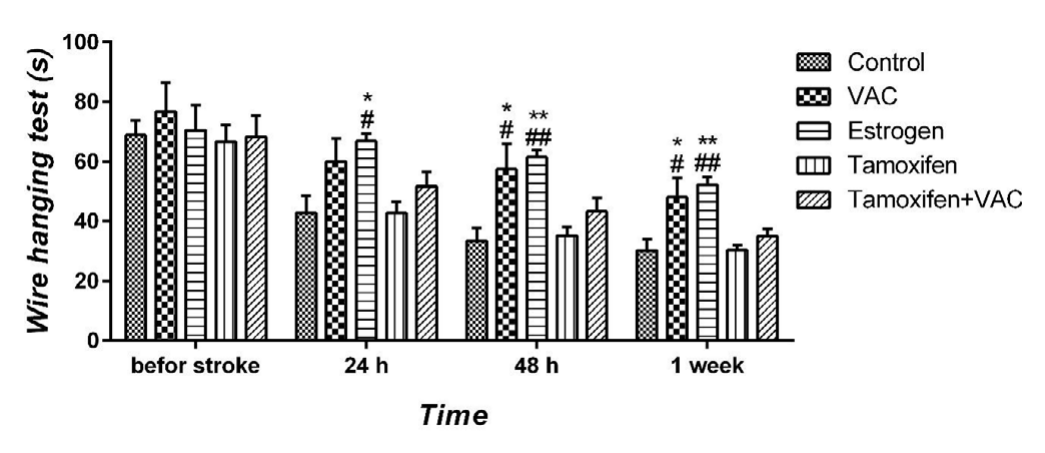
The effect of *Vitex agnus-castus* (VAC) extract, estrogen, tamoxifen, and the combination of tamoxifen plus VAC extract on grasping ability and forelimb strength of animals, which was measured by wire hanging test 24 hr, 48 hr, and one week after stroke. Data are presented as means±SEM. *P<0.05 and **P<0.01 vs control group. #*P*<0.05 and ##*P*<0.01 vs tamoxifen group

**Figure 4:**
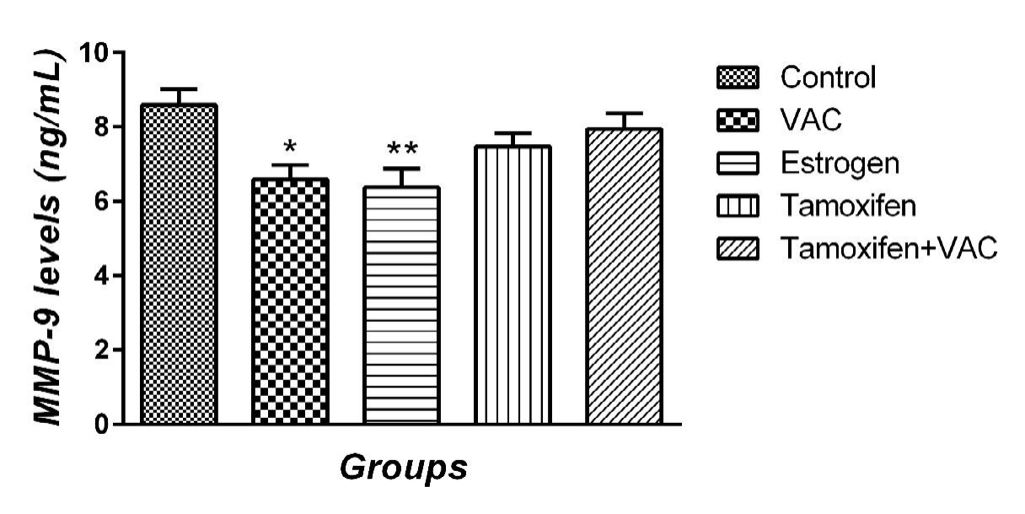
The effect of *Vitex agnus-castus* (VAC) extract, estrogen, tamoxifen, and the combination of tamoxifen plus VAC extract on matrix metalloproteinase-9 (MMP-9) levels. Data are presented as means±SEM. *P<0.05 and **P<0.01 vs control group

**Figure 5:**
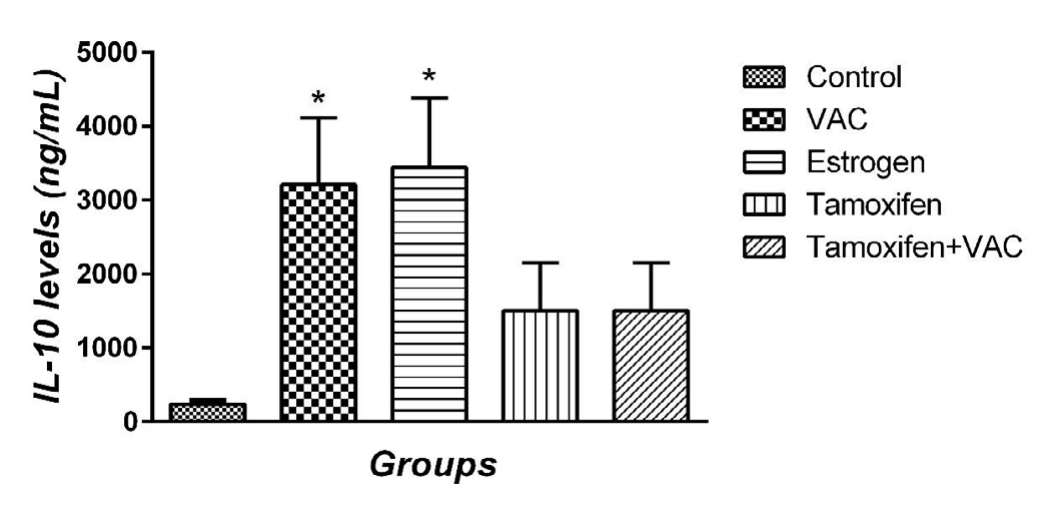
The effect of *Vitex agnus-castus* (VAC) extract, estrogen, tamoxifen, and the combination of tamoxifen plus VAC extract on interleukin-10 (IL-10) levels. Data are presented as means±SEM. *P<0.05 vs control group

*Vitex agnus-castus* (VAC) is one of these plants whose estrogenic effects is well documented in the literature (17). VAC extract is used as a homeopathic drug for the treatment of brain disorders (18). Phytochemicals such as essential oils, flavonoids, iridoids, and diterpenoids are found in VAC fruits (19). The major components of VAC in Iran are α-pinene, limonene, β-caryophyllene, sabinene, and β-farnesene (20). ER binding tests have identified that linoleic acid is the ER ligand of VAC extract (21). Also, it has been reported that apigenin as a flavonoid has high binding affinity to ER (22). Flavonoids and diterpenoids, isolated from the extract of VAC fruits, have been found to exhibit antioxidant and anti-inflammatory activity (17). Furthermore, numerous evidence in the literature has shown that VAC is safe and has no side effect or mortality, even at a high dose (5 g/kg) (20).

To the best of our search, the possible neuroprotective effect of VAC in stroke has not been previously studied. In the current study, we designed to determine whether the neuroprotection induced by dietary VAC extract is mediated through its estrogen agonistic property and/or its anti-inflammatory action. For doing this, we studied the effects of VAC both in the presence and in the absence of estrogen antagonist (tamoxifen) and examined the serum levels of IL-10 and MMP-9. 

## Materials and Methods

***Plant material and extraction method***

VAC seeds were collected from Esfahan province. The fruits were identified by experts of the Central Herbarium of Isfahan University with registration number 9252, in 2012. The fruits were dried in the air. Five hundred grams of the dried fruit was mixed with ethanol (50%) and extracted with Soxoleh appurtenance, and then solvent evaporation was performed by a rotary device. After that, it was dried at 50 ^°^C in the oven. In the end, the resulting powder was stored at -20 ^°^C until the experiment time. For administration, the frozen VAC extract was freshly dissolved in saline (23).

***Drugs***

Estradiol valerate (Alpha, Iran), as an ER agonist, was dissolved in saline (1 ml). Tamoxifen was used (Hormone-Alpha, Iran) as an antagonist of ER. Tamoxifen is a selective estrogen receptor modulator and has both agonist and antagonist properties in different organs. For example, in brain and breast block the ER and in bone and uterus stimulate these receptors. Tamoxifen was dissolved in saline (1 ml). 

***Animals***

A total number of 50 female mice (25–35 g) were fed food and water *ad libitum* (12-hr light-dark cycle). Mice were treated in accordance with the criteria presented in the Guide for Care and Use of Laboratory Animals (NIH US publication 86-23 revised 1985; http://oacu. od.nih.gov/regs/guide/guidex.htm). All experiments were approved by the Animal Ethics Committee of Rafsanjan University of Medical Science (permission no. 9/2330). 

***Ovariectomy***

Animals were anesthetized by intraperitoneal (IP) injection of xylazine and ketamine (4.5 mg/kg and 90 mg/kg). Then, mice were positioned ventrally and a ventral incision was made. Then the ovaries and the surrounding fat were sectioned by cauterization, the wound was closed, and penicillin (22,000 i.u, kg/day) was administered for two days after surgery. At the day of five after surgery, vaginal smears were taken daily for six consecutive days to verify the termination of hormonal cycles (24-26). 

***Experimental design***

Animals were randomly allocated to five groups based on the intervention as follows (n=10 in each group): 

1. Control: ovariectomized animals that were orally treated with 1 ml saline.

2. VAC: ovariectomized animals that were treated by VAC extract (80 mg/kg dissolved in 1 ml saline). 

3. Estrogen: ovariectomized animals that were treated with Estradiol valerate (40 µg/kg dissolved in 1 ml saline). 

4. Tamoxifen: ovariectomized animals that were treated with tamoxifen (100 µg/kg dissolved in 1 ml saline). 

4. Tamoxifen +VAC: ovariectomized animals that were treated with tamoxifen and VAC extract. 

The doses of hydro-alcoholic extract of VAC, Estradiol valerate, and tamoxifen were selected based on the previous investigations (23, 27). It should be noted that animals in all groups underwent the permanent middle cerebral artery occlusion (PMCAO) procedure and drugs were administered for four weeks prior to the stroke insult. 

***PMCAO induction***

Anesthesia was done by IP injection of ketamine and xylazine (90 mg/kg and 4.5 mg/kg). As previously reported, PMCAO induction was done by occlusion of the left MCA (28). Briefly, a small incision was made in the space between the left ear and eye. The temporal muscle was pulled laterally to expose the skull. Using a dental drill, a small puncture (1 mm diameter) was made on the temporal bone above the MCA. The dura matter was carefully retracted, then the MCA was cauterized permanently by a thermocoagulator. Finally, the temporal muscle was replaced, the skin was sutured and animals were returned to their individual cages.

***Behavioral assessment***

The sensorimotor function was assessed by the adhesive removal and wire hanging tests (28, 29). Animals were examined 24 hr before PMCAO, 24 hr, 48 hr, and one week after PMCAO. Briefly, a small adhesive tape (1 × 1 cm) was glued to the surface of the right forepaw, and the latency (in sec) to pull the tape from the right forepaw was recorded. The wire hanging test was done on a wire (1 mm diameter) that was stretched horizontally 50 cm above a cotton pad. The length of time (in sec) that the mice held onto the wire (latency) was recorded.

***Sample collection***

24 hr after the last behavioral test, animals were anesthetized with diethyl ether and blood samples were gathered by inducing a puncture in the left cardiac ventricle (30). The blood was centrifugated for 10 min at 3000 rpm and serum stored at -20 ^°^C until analysis. In the end, the brains of mice were isolated immediately.

***Measurement of the infarct volume, MMP-9, and IL-10***

We have previously reported the infarct volume measurement (31). In brief, after removing the brain form the skull, it was cut into 1 mm thick coronal sections and merged for 30 min in 2% 2,3,5 triphenyltetrazolium chloride (TTC; Sigma Chemical Co, St Louis, MO, USA) at 37 ^°^C. Then the sections were immersed in 10% formalin overnight. The infarct zone was analyzed using an image analyzer (Image J software, NIH Image, version 1.61 Bethesda, Maryland, USA). The total infarct area was calculated by adding the infarct areas of all sections and multiplied by the thickness of the brain sections to determine the infarct volume.

The serum levels of MMP-9 and IL-10 were measured according to the protocol of a commercial ELISA kit (R and D system, Minneapolis, MN, USA). 

***Statistical analysis***

GraphPad Prism software (version 6) was used for analysis of data (GraphPad Software San Diego, CA, USA). One-way ANOVA was used for analysis of data followed by Tukey’s *post hoc* test, and they were expressed as means±SEM. *P*<0.05 was accepted as a significant difference.

## Results

***Effect of VAC extract on infarct volume***

Infarct volume percentages are illustrated in Figure 1. No significant difference was observed between control and tamoxifen groups. Compared with the control and tamoxifen groups, VAC extract and estrogen groups decreased the infarct volume (*P*<0.05 and *P*<0.01, respectively). Co-administration of tamoxifen and VCA extract did not show any significant effect on infarct volume in comparison with control and tamoxifen groups. 

***Effect of VAC extract on adhesive removal test***

Time to remove the small adhesive tape from the forepaw of the injured hemisphere is presented in Figure 2. No significant difference between the control and tamoxifen animals in the sticky test was observed. VAC-treated animals exhibited a significant reduction in duration of sticky test one week (all *P*<0.05) after the PMCAO procedure compared with the control and tamoxifen groups. Also, estrogen-treated mice needed less time to remove the label 48 hr (all *P*<0.05) and one week (all *P*<0.01) after PMCAO procedure compared with control and tamoxifen groups. In addition, co-administration of tamoxifen and VCA extract had no significant effect on the sticky test compared with control and tamoxifen groups.

***Effect of VAC extract on wire hanging test***

Grasping ability and forelimb strength after ischemia was measured by the wire hanging test. There was no significant difference between the control and tamoxifen animals in hanging time. The latency of fall was significantly enhanced in the VAC extract group 48 hr (all *P*<0.05) and one week after PMCAO (all *P*<0.05) compared with the control and tamoxifen groups (Figure 3). Also, the duration of the hanging test significantly increased in estrogen-treated animals 24 hr (all *P*<0.05), 48 hr (all *P*<0.01), and one week after MCAO (all *P*<0.01) compared with control and tamoxifen groups. In addition, co-administration of tamoxifen and VCA extract had no significant effect on hanging time compared with control and tamoxifen groups.

***Effect of VAC extract on MMP-9 and IL-10 serum levels***

MMP-9 level was measured and results are illustrated in Figure 4. No significant difference was observed between the control and tamoxifen animals in MMP-9 serum levels. VAC extract and estrogen groups decreased MMP-9 serum level compared with the control group one week after MCAO (*P*<0.05 and *P*<0.01, respectively). Also, the serum level of IL-10 was investigated (Figure 5). No significant difference between the control and tamoxifen animals in the serum level of IL-10 was observed. VAC extract and estrogen groups significantly increased the levels of IL-10 compared with the control group one week after the stroke (all *P*<0.05). In addition, co-administration of tamoxifen and VCA extract had no significant effect on the serum levels of MMP-9 and IL-10 compared with control and tamoxifen groups.

## Discussion

Our results demonstrated that administration of VAC extract ameliorates the deleterious effects of cerebral ischemia by reducing the infarct volume and sensory-motor disorder as well as MMP-9 level and increasing the serum level of IL-10. 

Our results indicated that VAC extract alone, similar to estrogen, reduced the infarct volume. On the other hand, co-administration of tamoxifen and VCA extract did not reduce infarct volume. Our data are in line with the results of previous reports that demonstrated the protective effects of phytoestrogens in different models of strokes. A study indicated that the infarct size shrinks significantly in the focal stroke, in animals that received only two weeks of soy-rich diet compared with animals that were fed the low-soy diet (32). Researchers have demonstrated that phytoestrogens treatment in cerebral ischemia reduces the oxidative stress and neuronal degeneration (13). Wang *et al*. have shown that Biochanin A, a natural isoflavonoid classified as a phytoestrogen, has neuroprotective effects on cerebral ischemia/reperfusion in rats (33). 

Adhesive removal and wire hanging behavioral tests indicated that VAC extracts like estrogen improve behavioral functions. In addition, co-administration of tamoxifen and VCA extract has no effects on sticky and wire-hanging tests. There is little research review showing the effect of phytoestrogens on neurological deficits after stroke. In parallel with our findings, Huang *et al*. have suggested that phytoestrogens improve neurological performances in temporary middle cerebral artery occlusion (34).

Matrix Metalloproteinases (MMPs) have roles in the pathogenesis of cerebral ischemia such as inflammation, disruption of brain blood barrier (BBB), and oxidative stress (28). It has been reported that estrogen may prevent BBB disruption through MMPs inhibition in focal cerebral ischemia (35). Our data showed that the MMP-9 level is increased after the induction of PMCAO. Moreover, the MMP-9 level was declined in ischemic animals treated with VAC extract or estrogen. Furthermore, co-administration of tamoxifen and VCA extract has no effects on MMP-9 level. Thus, it can be suggested that vitex extract also contributes to the reduction of MMP-9 level and can prevent BBB disruption by affecting estrogen receptors. However, this has to be demonstrated by further studies.

We also examined the level of the anti-inflammatory cytokine, IL-10. VAC extract significantly increased the level of IL-10. This effect was similar to estrogen. Moreover, co-administration of tamoxifen and VAC extract has no effects on IL-10 level. This finding also confirms that VAC extract increases the level of IL-10 through its estrogenic properties. The effect of phytoestrogens on IL-10 has already been reported and in agreement with our finding genistein (a phytoestrogen) regulates the expression of IL-10 by stimulated murine splenocytes (36). 

## Conclusion

To the best of our knowledge, this is the first investigation that evaluated the use of VAC in reducing the deleterious outcomes of the PMCAO model. Based on our current findings and other investigators’ findings, VAC has neuroprotective effects and can reduce stroke injuries by estrogenic and anti-inflammatory effects. Thus, VAC extract can be taken into account as a potential complementary therapeutic compound in the treatment of stroke in menopause.

## References

[1] Prongay KD, Lewis AD, Hurn PD, Murphy SJ (2010). Dietary soy may not confound acute experimental stroke infarct volume outcomes in ovariectomized female rats. Lab Anim.

[2] Liu R, Wen Y, Perez E, Wang X, Day AL, Simpkins JW (2005). 17β-Estradiol attenuates blood–brain barrier disruption induced by cerebral ischemia–reperfusion injury in female rats. Brain Res.

[3] Merchenthaler I, Dellovade TL, Shughrue PJ (2003). Neuroprotection by estrogen in animal models of global and focal ischemia. Ann N Y Acad Sci.

[4] Habib P, Dreymueller D, Ludwig A, Beyer C, Dang J (2013). Sex steroid hormone-mediated functional regulation of microglia-like BV-2 cells during hypoxia. J Steroid Biochem Mol Biol.

[5] Zhang J, Lapato A, Bodhankar S, Vandenbark AA, Offner H (2015). Treatment with IL-10 producing B cells in combination with E2 ameliorates EAE severity and decreases CNS inflammation in B cell-deficient mice. Metab Brain Dis.

[6] Protti GG, Gagliardi RJ, Forte WC, Sprovieri SR (2013). Interleukin-10 may protect against progressing injury during the acute phase of ischemic stroke. Arq Neuropsiquiatr.

[7] Viscoli CM, Brass LM, Kernan WN, Sarrel PM, Suissa S, Horwitz RI (2001). A clinical trial of estrogen-replacement therapy after ischemic stroke. N Engl J Med.

[8] Duncan AM, Phipps WR, Kurzer MS (2003). Phyto-oestrogens. Best Pract Res Clin Endocrinol Metab.

[9] Sirotkin AV, Harrath AH (2014). Phytoestrogens and their effects. Eur J Pharmacol.

[10] Yaman M EO, Cosar M, Bas O, Sahin O, Mollaoglu H (2007). Oral administration of avocado soybean unsaponifiables (ASU) reduces ischemic damage in the rat hippocampus. Arch Med Res.

[11] Castelló-Ruiz M TG, Burguete M, Salom J, Gil J, Miranda F (2011). Soy-derived phytoestrogens as preventive and acute neuroprotectors in experimental ischemic stroke: influence of rat strain. Phytomedicine.

[12] Soni M, Rahardjo TB, Soekardi R, Sulistyowati Y, Lestariningsih, Yesufu-Udechuku A (2014). Phytoestrogens and cognitive function: a review. Maturitas.

[13] Aras AB, Guven M, Akman T, Alacam H, Kalkan Y, Silan C (2015). Genistein exerts neuroprotective effect on focal cerebral ischemia injury in rats. Inflammation.

[14] E Orhan I, F Nabavi S, Daglia M, C Tenore G, Mansouri K, M Nabavi S (2015). Naringenin and atherosclerosis: A review of literature. Curr Pharm Biotechnol.

[15] Kashtriya R, Shaikh Y, Nazeruddin G (2015). A Brief Review: Flavonoids as a pharmacophore. J Applicable Chem.

[16] Schreihofer DA, Deutsch C, Lovekamp-Swan T, Sullivan JC, Dorrance AM (2010). Effect of high soy diet on the cerebrovasculature and endothelial nitric oxide synthase in the ovariectomized rat. Vascul Pharmacol.

[17] Rani A, Sharma A (2013). The genus Vitex: A review. Pharmacognosy reviews.

[18] Marotta F, Mao G, Liu T, Chui D, Lorenzetti A, Xiao Y (2006). Anti-inflammatory and neuroprotective effect of a phytoestrogen compound on rat microglia. Ann N Y Acad Sci.

[19] Ghannadi A, Bagherinejad M, Abedi D, Jalali M, Absalan B, Sadeghi N (2012). Antibacterial activity and composition of essential oils from Pelargonium graveolens L’Her and Vitex agnus-castus L. Iran J Microbiol.

[20] Khalilzadeh E, Vafaei Saiah G, Hasannejad H, Ghaderi A, Ghaderi S, Hamidian G (2015). Antinociceptive effects, acute toxicity and chemical composition of Vitex agnus-castus essential oil. Avicenna J Phytomed.

[21] Liu J, Burdette J, Sun Y, Deng S, Schlecht S, Zheng W (2004). Isolation of linoleic acid as an estrogenic compound from the fruits of Vitex agnus-castus L(chaste-berry). Phytomedicine.

[22] Chen SN, Friesen JB, Webster D, Nikolic D, van Breemen RB, Wang ZJ (2011). Phytoconstituents from Vitex agnus-castus fruits. Fitoterapia..

[23] Honari N, Pourabolli I, Hakimizadeh E, Roohbakhsh A, Shamsizadeh A, Vazirinejad R (2012). Effect of Vitex agnus-castus extraction on anxiety-like behaviors in ovariectomized rats. J Babol Univ Med Sci.

[24] Fatemi I, Heydari S, Kaeidi A, Shamsizadeh A, Hakimizadeh E, Khaluoi A (2018). Metformin ameliorates the age-related changes of d-galactose administration in ovariectomized mice. Fundam Clin Pharmacol..

[25] Fatemi I, Delrobaee F, Bahmani M, Shamsizadeh A, Allahtavakoli M (2019). The effect of the anti-diabetic drug metformin on behavioral manifestations associated with ovariectomy in mice. Neurosci Lett.

[26] Delrobaei F, Fatemi I, Shamsizadeh A, Allahtavakoli M (2018). Ascorbic acid attenuates cognitive impairment and brain oxidative stress in ovariectomized mice. Pharmacol Rep.

[27] Sharma K, Mehra RD (2008). Long-term administration of estrogen or tamoxifen to ovariectomized rats affords neuroprotection to hippocampal neurons by modulating the expression of Bcl-2 and Bax. Brain Res.

[28] Allahtavakoli M, Amin F, Esmaeeli-Nadimi A, Shamsizadeh A, Kazemi-Arababadi M, Kennedy D (2015). Ascorbic acid reduces the adverse effects of delayed administration of tissue plasminogen activator in a rat stroke Model. Basic Clin Pharmacol Toxicol.

[29] Hadadianpour Z, Fatehi F, Ayoobi F, Kaeidi A, Shamsizadeh A, Fatemi I (2017). The effect of orexin-A on motor and cognitive functions in a rat model of Parkinson’s disease. Neurol Res.

[30] Hassanshahi A, Shafeie SA, Fatemi I, Hassanshahi E, Allahtavakoli M, Shabani M (2017). The effect of Wi-Fi electromagnetic waves in unimodal and multimodal object recognition tasks in male rats. Neurol Sci.

[31] Hakimizadeh E, Shamsizadeh A, Roohbakhsh A, Arababadi MK, Hajizadeh MR, Shariati M (2017). TRPV1 receptor-mediated expression of toll-like receptors 2 and 4 following permanent middle cerebral artery occlusion in rats. Iran J Basic Med Sci.

[32] Schreihofer DA, Do KD, Schreihofer AM (2005). High-soy diet decreases infarct size after permanent middle cerebral artery occlusion in female rats. Am J Physiol Regul Integr Comp Physiol.

[33] Wang W, Tang L, Li Y, Wang Y (2015). Biochanin A protects against focal cerebral ischemia/reperfusion in rats via inhibition of p38-mediated inflammatory responses. J Neurol Sci.

[34] Huang G, Cao X, Zhang X, Chang H, Yang Y, DU W (2009). Effects of soybean isoflavone on the notch signal pathway of the brain in rats with cerebral ischemia. J Nutr Sci Vitaminol.

[35] Dong X, Song Y-N, Liu W-G, Guo X-L (2009). Mmp-9, a potential target for cerebral ischemic treatment. Current neuropharmacology.

[36] Rachoń D, Rimoldi G, Wuttke W (2006). In vitro effects of genistein and resveratrol on the production of interferon-γ (IFN-γ) and interleukin-10 (IL-10) by stimulated murine splenocytes. Phytomedicine.

